# Association of Diverticulitis with Prolonged Spondyloarthritis: An Analysis of the ASAS-COMOSPA International Cohort

**DOI:** 10.3390/jcm8030281

**Published:** 2019-02-26

**Authors:** Mohammad H. Derakhshan, Nicola J. Goodson, Jonathan Packham, Raj Sengupta, Anna Molto, Helena Marzo-Ortega, Stefan Siebert, BRITSpA and the ASAS-COMOSPA Investigators

**Affiliations:** 1 Institute of Infection, Immunity and Inflammation, University of Glasgow, Glasgow G12 8TA, UK; 2 Academic Rheumatology Department, University of Liverpool, Liverpool L9 7AL, UK; Nicola.Goodson@liverpool.ac.uk; 3 Haywood Rheumatology Centre, Staffordshire ST6 7AG, UK; Jon.Packham@mpft.nhs.uk; 4 Royal National Hospital for Rheumatic Diseases, Bath BA1 1RL, UK; Rajsen99@gmail.com; 5 Paris Descartes University, Hôpital Cochin, 75005 Paris, France; Anna.Molto@aphp.fr; 6 NIHR LBRC, Leeds Teaching Hospitals Trust and LIRMM, University of Leeds, Leeds LS7 4SA, UK; H.Marzo-Ortega@leeds.ac.uk

**Keywords:** spondyloarthropathies, ankylosing spondylitis, diverticulitis, epidemiology, comorbidity, disease duration, delay in diagnosis

## Abstract

This study examined the relationship between spondyloarthritis (SpA) duration and gastrointestinal comorbidities other than inflammatory bowel disease (IBD). We evaluated the association between SpA duration and upper gastrointestinal ulcers, hepatitis B (HBV), hepatitis C (HCV) and diverticulitis using data from a large international cross-sectional study. Binary regression models were created, adjusted for age, sex, body mass index (BMI), smoking, alcohol, non-steroidal anti-inflammatory drugs (NSAIDs), disease-modifying anti-rheumatic drugs (DMARDs), biologics, steroids, IBD history and country. Subgroup analysis was performed by disease phenotype. The data of 3923 participants were analysed. The prevalence of gastrointestinal conditions were 10.7% upper gastrointestinal ulcers; 4.7% viral hepatitis and 1.5% diverticulitis. While SpA duration was not associated with upper gastrointestinal ulcers, HBV or HCV, longer SpA duration was significantly associated with diverticulitis (odds ratios (OR) = 1.18, 95% confidence interval (CI): 1.03–1.34), reflecting an 18% increase for every five years of SpA duration. Other significant associations with diverticulitis were age and high alcohol intake but not medication history. In subgroup analyses, the association was strongest with those with axial SpA. The reasons for this association of increased diverticulitis with disease duration in SpA, especially those with axial disease, are unclear but may reflect shared underlying gut inflammation. Diverticulitis should be considered, in addition to IBD, when SpA patients present with lower gastrointestinal symptoms.

## 1. Introduction

Spondyloarthritis (SpA) represents a group of inflammatory rheumatic musculoskeletal disorders with shared pathogeneses, clinical and therapeutic features. In addition to the characteristic extra-articular manifestations, such as acute anterior uveitis, psoriasis and inflammatory bowel disease (IBD) that characterise these conditions [1,2], they are also associated with a number of comorbidities.

The prototypic gastrointestinal association with SpA is IBD, which defines enteropathic arthritis. Musculoskeletal clinical features consistent with SpA have been reported to occur in up to 13% of patients with IBD [3]. Furthermore, 6.8% of people with ankylosing spondylitis (AS) develop IBD [4], while microscopic signs of gut inflammation were observed in over 60% of patients with SpA [5].

People with SpA are also at increased risk of other gastrointestinal effects as a result of their treatments. Upper gastrointestinal ulcers are commonly reported comorbidities, mainly attributed to the chronic intake of non-steroidal anti-inflammatory drugs (NSAIDs) and use of steroids in SpA [6,7], although the introduction of selective cox-2 inhibitors and concomitant gastroprotection have relatively declined their incidence [8,9]. The use of tumour necrosis factor (TNF)-blockers in the treatment of SpA is associated with an increased risk of reactivation of latent viral hepatitis [10,11]. Therefore, gastrointestinal conditions other than IBD are important comorbidities in SpA, which may contribute to morbidity and have implications for therapeutic decisions in SpA.

In this study, we evaluate the association of gastrointestinal conditions with SpA in more detail by exploring the potential associations between the chronology of SpA, particularly “SpA disease duration”, and the prevalence of gastrointestinal comorbidities using data from the Assessment of Spondyloarthritis international Society-COMOrbidities in SPondyloArthritis (ASAS-COMOSPA) study.

## 2. Methods and Materials

### 2.1. Study Design

The current manuscript presents a comprehensive analysis of gastrointestinal comorbidities recorded in the large global cross-sectional ASAS-COMOSPA study. Detailed methodologies of the original study, which was conducted between January 2010 to September 2014, has been published elsewhere [12,13].

Briefly, participating rheumatology centres in 22 countries recruited consecutive adult patients with a diagnosis of SpA based on the ASAS criteria (axial or peripheral involvement) [14,15]. During a face-to-face interview with consenting participants, pre-specified information was obtained, and medical records were reviewed to validate the information. Specific questions targeted SpA history, treatments, extra-articular manifestations, comorbidities and lifestyle factors. In addition to general demographic data, including age and gender, lifestyle variables relevant to gastrointestinal disease including current body mass index (BMI), smoking status and alcohol intake (past and current) were recorded. Past and current medications (NSAIDs, corticosteroids, conventional and biological disease-modifying anti-rheumatic drugs (DMARDs)) were also recorded. Details of SpA disease phenotypes such as axial involvement, peripheral arthritis, enthesitis, dactylitis and extra-articular manifestations were collected.

In addition to the study visit date, the date of diagnosis, and, where relevant, date(s) of first musculoskeletal symptoms (back pain, peripheral joint symptoms, enthesitis or dactylitis) were captured. The following gastrointestinal comorbidities were recorded: ever diagnosis of upper gastrointestinal ulcers, diverticulitis, hepatitis B (HBV) and hepatitis C (HCV).

### 2.2. Ethical Considerations

As described in the original ASAS-COMOSPA study [12], in each participant country, consecutive adult patients with SpA who were able to understand, and complete questionnaires were included. Local ethical approval was obtained in all participating countries and the study was conducted according to guidelines for good clinical practice. Written informed consent was obtained from all subjects before enrolment.

### 2.3. Data Analysis

Central tendencies in groups are presented as median and interquartile range (IQR), unless otherwise stated. Where necessary, the difference between independent groups was examined using the Mann Whitney U test. In order to examine the association between SpA chronology and prevalence of gastrointestinal co-morbidities, we defined “SpA Disease Duration” as the period between the date when SpA was formally diagnosed and the date of the study. The “Delay in SpA Diagnosis” was defined as the time interval between the first musculoskeletal symptoms of SpA and the confirmed diagnosis of SpA.

The association between SpA disease duration (defined in 5-year blocks) and gastrointestinal comorbidities was examined using uni-variable and multi-variable logistic regression. Each model included one gastrointestinal comorbidity (e.g., ever diagnosis of HBV hepatitis) as dependent and SpA disease duration as predictor, adjusted for relevant confounders. Common adjusting variables in all models were age (continuous), sex (reference: females), current BMI (continuous), history of smoking (pack-years), alcohol (reference: non-drinker), ever use of NSAIDs (reference: none), ever use of steroids (reference: none), ever use of synthetic DMARDs (reference: none), use of biological DMARDs (reference: none) and country of residence. Models evaluating the association between disease duration and diverticulitis were adjusted for history of IBD. Additional adjusting factors were added to individual models if biologically plausible explanations were present. The potential issue of collinearity was examined using correlation matrix, tolerance and variation inflation factors in a linear regression model. The magnitude of the associations was presented using Wald statistics, odds ratios (OR) and relevant 95% confidence intervals (CI). All significance levels were set to a *p* value less than 0.05.

For subgroup analyses, the entire cohort was categorised into subgroups on the basis of having axial, peripheral or mixed axial-peripheral musculoskeletal involvement. Axial was defined as the clinicians’ report of “ever suffered from inflammatory chronic (at least 3 months) back pain starting before the age of 45 years”, while peripheral was defined as the presence of “ever suffered from peripheral joint disease” or “symptoms suggestive of enthesitis/dactylitis”.

## 3. Results

### 3.1. Basic Characteristics of Cohort

The data of 3923 participants were available for analysis. Only 51 participants were excluded, comprising 41 aged under 18 years, 5 with missing date of visit, 4 with missing date of birth and 1 with missing information of both date of birth and date of visit. Participant age ranged from 18 to 100, with a median (IQR) of 42.0 (32.0–53.0) years; 64.9% were male. Median BMI was 25.3 (IQR: 22.5–28.7). Almost a quarter (23.0%) of the participants were current smokers, 23.4% were ex-smokers and 53.6% had never smoked; 5.3% of total cohort had history of IBD.

The median (IQR) age at SpA diagnosis was 33.0 (25.0–43.0) years while the median (IQR) age at which the first musculoskeletal symptom(s) of SpA appeared was 29.4 (21.9–39.9) years. The estimated median (IQR) SpA disease duration was 5.1 (1.3–11.8) years and the estimated median (IQR) delay in SpA diagnosis was 1.1 (0.0–5.9) years.

### 3.2. Gastrointestinal Ulcers and SpA Disease Duration

Peptic ulcers were the most commonly reported gastrointestinal comorbidities in the COMOSPA study, with 418 cases affecting 10.7% of participants. The participants with “ever diagnosis of peptic ulcers” had a median (IQR) age of 48.0 (36.0–57.0) and equal gender distribution, with a male/female ratio of 0.9:1.

The probability of having a recorded diagnosis of gastric or duodenal ulcers in participants with SpA was slightly increased with longer SpA disease duration in the univariable model (OR = 1.06, 95% CI: 1.01–1.12; *p* = 0.031) (Table 1). This association was no longer present after adjustment for potential confounders (OR = 1.04, 95% CI: 0.97–1.11; *p* = 0.330). Other factors predicting the ever diagnosis of upper gastrointestinal ulcers were age (OR = 1.03, 95% CI: 1.02–1.04; *p* < 0.001) and ever intake of steroids (OR = 1.48, 95% CI: 1.12–1.95; *p* = 0.006). There was no association between delay in SpA diagnosis and ever diagnosis of upper gastrointestinal ulcers (Table 1).

### 3.3. Diverticulitis and SpA Disease Duration

In total, there were 58 participants with SpA in the study with an ever diagnosis of diverticulitis, which represent 1.5% of the 3872 responders. These participants had a median (IQR) age of 57.5 (50.8–65.3) years and a male/female ratio of 0.7:1.

The chance of having a recorded diagnosis of diverticulitis in participants with SpA increased with increasing SpA disease duration in the univariable model (OR = 1.35, 95% CI: 1.23–1.49; *p* < 0.001). As shown in Table 2, the association was evident even after adjustment for age, history of IBD and other potential confounders. The risk of developing diverticulitis increased 18% for every 5 years of SpA disease duration, which was statistically robust (OR = 1.18, 95% CI: 1.03–1.34; *p* = 0.016).

As shown in the results of multivariable analysis, apart from SpA disease duration, only age (OR = 1.06, 95% CI: 1.03–1.08; *p* < 0.001) and high alcohol (≥3 units per day) intake (OR = 4.21, 95% CI: 1.56–11.38; *p* = 0.005) were significant predictors of diverticulitis. Other parameters including gender, BMI, drug history (NSAIDs, steroids, DMARDs and biologics) and ever diagnosis of IBD did not demonstrate any association with ever diagnosis of diverticulitis. There was no association between delay in SpA diagnosis and ever diagnosis of diverticulitis (Table 2).

### 3.4. Diverticulitis and SpA Subgroups

When the association between diverticulitis and SpA disease duration was examined across SpA subgroups, participants with “any axial dominant phenotype” showed the strongest association (OR = 1.23, 95% CI: 1.06–1.43; *p* = 0.006), while the subgroup with “any peripheral phenotype” had the weakest link (OR = 1.18, 95% CI: 1.01–1.38; *p* = 0.038). The subgroups with “axial-only” or “peripheral-only” phenotypes did not demonstrate any association between SpA disease duration and a diagnosis of diverticulitis, although the number of participants in those subgroups was small (Table 3). There were no associations between delay in SpA diagnosis and a diagnosis of diverticulitis in the SpA subgroups (Table 3).

### 3.5. Viral Hepatitis and SpA Disease Duration

There were 178 participants with SpA in the study who had a recorded diagnosis of viral hepatitis, comprising 132 (3.5%) with HBV and 46 (1.2%) with HCV. The median (IQR) age for HBV was 42.5 (31.0–54.0) years and for HCV was 49.0 (40.3–57.0) years. The risk of the infection was similar in males and females for both HBV (male/female = 1.4:1, *p* = 0.094) and HCV (male/female = 1:1, *p* = 1.000) infections. There was no association between ever diagnosis of HBV and SpA disease duration in either the univariable or multivariable analysis. The only factor demonstrating significant association with ever diagnosis of HBV was alcohol intake of 3 or more units per day (OR = 2.79, 95% CI: 1.11–7.04; *p* = 0.029). There was no association between delay in SpA diagnosis and ever diagnosis of HBV (Table 4).

Similarly, there was no association between ever diagnosis of HCV and SpA disease duration in either the univariable or multivariable analysis. The only factor indicating a significant association with ever diagnosis of HCV was age (OR = 1.07, 95% CI: 1.04–1.11, *p* < 0.001). There was no association between delay in SpA diagnosis and ever diagnosis of HCV (Table 5).

## 4. Discussion

To the best of our knowledge, this is the first report of increased risk of diverticulitis with increasing disease duration in patients with SpA. The risk was approximately 18% for every 5 years of SpA disease duration and persisted even after adjustment for potential confounders including age.

Diverticulosis, the presence of multiple diverticula, of the lower gastrointestinal tract is a relatively common finding in elderly people undergoing radiologic and endoscopic examinations for various reasons. Its prevalence appears to have increased during recent decades, although those reports rely on diagnosis in people who had an investigation for lower gastrointestinal complaints, so might reflect increased numbers undergoing investigations rather than a true increasing prevalence trend among the general population [16,17].

Unlike the largely asymptomatic nature of diverticulosis, diverticulitis implies the presence of inflamed or infected diverticula and is a serious clinical entity with significant associated morbidity. People with acute diverticulitis are usually unwell with abdominal pain, often in the left lower quadrant, fever and localised peritonitis. In terms of the epidemiological characteristics, diverticulitis is far less common than diverticulosis [18,19], although the medical literature contains more information about its incidence rather than its prevalence due to the acute nature of diverticulitis.

The development of diverticular disease in general and diverticulitis in particular is associated with a number of lifestyle and medical conditions. Older age and obesity are the strongest risk factors, while physical inactivity is reported to be associated with higher risk of diverticulitis and diverticular bleeding [18]. These risk factors may generally predispose individuals to chronic comorbidities with shared pathogenic pathways. Advanced age was associated with higher risk of diverticulitis in our study, but the study was unable to demonstrate a similar association with BMI.

IBD is a well-recognized extra-articular manifestation of SpA and was therefore examined as a potential confounding factor. Our data did not indicate any association between IBD and diverticulitis in this cross-sectional SpA cohort. While it was not recorded in our study how the diagnosis of diverticulitis and IBD was made, it is unlikely that these conditions were confused as data was captured using both a patient questionnaire and review of the medical notes. In addition to the diagnosis, the medical notes would also have included relevant investigations, usually CT imaging for acute diverticulitis and colonoscopy and biopsy for IBD.

In the absence of obvious lifestyle or medical conditions shared between SpA and diverticulitis, speculation about the background pathophysiology is difficult. Furthermore, a questionnaire-based, cross-sectional study, even if validated by medical records, cannot assess all factors involved in the disease pathway or presentation. People with SpA are known to be at increased risk of both IBD and asymptomatic microscopic gut inflammation [5], so there may be shared mechanisms with diverticulitis. Microscopic colitis, comprising collagenous and lymphocytic colitis, is present on endoscopy in up to 50% of people with SpA and is associated with more severe disease [20,21]. It is unlikely that the data capture methods in our study would be sufficient to capture microscopic colitis without additional laboratory or endoscopic investigations. Moreover, antibodies associated with IBD have been reported in more than half of AS patients without signs or symptoms of IBD, while alterations in the gut microbiome are increasingly implicated across the SpA spectrum [22,23]. The strongest reported association of IBD with SpA is in those with axial disease [5]; interestingly, in our study, the strongest association of diverticulitis with SpA disease duration was seen in those participants with axial disease. Taken together, these findings support the hypothesis of chronic gut inflammation associated with SpA, which may reflect shared pathophysiology or put people with SpA at increased risk of diverticulitis. More detailed studies are necessary to confirm our findings and this hypothesis. However, the clinical implication is that diverticulitis should be considered, in addition to IBD, in patients with SpA who present with lower gastrointestinal symptoms, particularly in the elderly, those with longer SpA disease duration and those who drink ≥3 units of alcohol per day. It should be noted that the cross-sectional nature of this study in a selected group of patients with SpA means that no comment can be made regarding the overall prevalence in comparison to the general population.

Upper gastrointestinal ulcers, comprising gastric and duodenal ulcers, were common diagnoses in general clinical practice until recent decades. The steadily decreasing trend in upper gastrointestinal ulcer prevalence is attributable to a sharp decrease in the prevalence of Helicobacter pylori infection globally [24,25], in addition to increased use of gastro-protection. Upper gastrointestinal ulcers are of particular interest in SpA due to the use of corticosteroids and particularly NSAIDs in the treatment of SpA [7,26]. Chronic use of these medicines predisposes patients to upper gastrointestinal ulcers through breakage in the mucosal defence mechanisms against gastric acid, bile and proteolytic enzymes, although eradication of H.pylori infection and concomitant use of proton pump inhibitors or other gastro-protective compounds have significantly reduced the risk of ulcers with these agents [27,28]. There was no excess risk of upper gastrointestinal ulcers in our participants with longer disease duration of SpA when corrected for confounders, suggesting that any increased risk of upper gastrointestinal ulcers is not related to SpA directly but more likely associated with medications. A significant association was seen with ever use of steroids, but not NSAIDs. It should however be noted that the vast majority of our cohort had received NSAIDs at some stage since the onset of the disease, affecting the ability to detect this association, while those with upper gastrointestinal symptoms are likely to have stopped their NSAIDs.

There is considerable literature on the reactivation of latent HBV or HCV virus during anti-TNF therapy for SpA [10,11] and current treatment guidelines accordingly recommend serologic screening before commencing any biologic agents [29]. We found no associations between SpA disease duration and ever diagnosis of either HBV or HCV, suggesting there is no association between SpA and these conditions. In fact, it is worth noting that a number of studies have reported a protective role for HLA-B27 in HCV infection through various immunological mechanisms [30].

## 5. Conclusions

In summary, using data from the large global ASAS-COMOSPA cohort, we were able to demonstrate an unexpected and previously unreported association of diverticulitis with SpA disease duration. This lends further support to the key role of the intestine in SpA beyond the well-recognised association with IBD. Reassuringly, the risk of upper gastrointestinal ulcers did not increase with SpA disease duration in this large cohort, suggesting that any association may be likely mainly related to pharmacotherapies, particularly NSAIDs and steroids, rather than a direct consequence of the underlying SpA. Consistent with current treatment recommendations for SpA [31,32], clinicians managing these patients should actively consider gastrointestinal symptoms and comorbidities, and minimise the risk of upper gastrointestinal symptoms associated with medications. Furthermore, diverticulitis should be considered in the differential diagnosis, in addition to IBD, in people with SpA who present with lower gastrointestinal symptoms, particularly the elderly, those with longer SpA disease duration, axial involvement and those with increased alcohol intake.

## Figures and Tables

**Table 1:** Risk of upper gastrointestinal ulcers associated with longer disease duration in participants with spondyloarthritis (SpA).

Factors & Covariates	Wald	*p-*Value	OR	95% CI for OR
Univariable				
SpA Disease Duration (5-year blocks)	4.662	0.031	1.060	1.005–1.118
Multivariable				
**SpA Disease Duration (5-year blocks)**	**0.950**	**0.330**	**1.035**	**0.965–1.110**
**Delay in SpA Diagnosis**	**0.012**	**0.911**	**0.999**	**0.985–1.014**
Age (year)	31.007	<0.001	1.029	1.019–1.039
Gender (reference: Female)	0.123	0.726	1.046	0.812–1.347
Current BMI	0.002	0.965	1.000	0.979–1.022
Smoking (pack-year)	0.002	0.963	1.000	0.992–1.008
Alcohol (reference: Never)	2.198	0.532		
Ex-drinker	2.033	0.154	1.414	0.878–2.278
Current, <3 units/day	0.537	0.464	1.130	0.815–1.565
Current, ≥3 units/day	0.509	0.475	1.213	0.713–2.065
Ever use of NSAIDs	0.080	0.777	1.071	0.666–1.721
Ever use of Steroids	7.663	0.006	1.476	1.120–1.945
Ever use of Synthetic DMARDs	1.560	0.212	1.193	0.905–1.573
Ever use of Biologic DMARDs	1.516	0.218	1.165	0.914–1.486
Country-based variation	235.039	<0.001		

Note: SpA: spondyloarthritis; BMI: body mass index; NSAID; non-steroidal anti-inflammatory drugs; DMARDs: disease modifying anti-rheumatic drugs. OR: Odds Ratio; CI: Confidence Interval.

**Table 2:** Risk of diverticulitis associated with longer disease duration in participants with SpA.

Factors & Covariates	Wald	*p-*Value	OR	95% CI for OR
Univariable				
SpA Disease Duration (5-year blocks)	38.492	<0.001	1.351	1.229–1.486
Multivariable				
**SpA Disease Duration (5-year blocks)**	**5.805**	**0.016**	**1.176**	**1.031–1.342**
Delay in SpA Diagnosis	1.278	0.258	1.016	0.988–1.044
Age (year)	19.263	<0.001	1.055	1.030–1.081
Gender (reference: Female)	3.752	0.053	0.545	0.295–1.007
Current BMI	0.011	0.916	0.997	0.946–1.051
Smoking (pack-year)	1.886	0.170	1.012	0.995–1.029
Alcohol (reference: Never)	8.534	0.036		
Ex-drinker	0.045	0.832	1.161	0.293–4.595
Current, <3 units/day	2.609	0.106	1.923	0.870–4.251
Current, ≥3 units/day	8.044	0.005	4.213	1.559–11.381
Ever use of NSAIDs	1.337	0.248	0.569	0.219–1.480
Ever use of Steroids	1.211	0.271	1.438	0.753–2.745
Ever use of Synthetic DMARDs	0.382	0.536	0.820	0.438–1.537
Ever use of Biologic DMARDs	0.016	0.898	1.040	0.569–1.902
History of IBD	0.000	1.000	1.000	0.332–3.010
Country-based variation	11.716	0.947		

Note: SpA: spondyloarthritis; BMI: body mass index; NSAID; non-steroidal anti-inflammatory drugs; DMARDs: disease modifying anti-rheumatic drugs; IBD: inflammatory bowel diseases.

**Table 3:** Summary of association between risk of diverticulitis and either “SpA disease duration” or “Delay in SpA Diagnosis”, adjusted for all potential confounders.

Subgroup	SpA Disease Duration			Delay in SpA Diagnosis		
	*p*-Value	OR	95% CI for OR	*p*-Value	OR	95% CI for OR
All participants	0.016	1.176	1.031–1.342	0.258	1.016	0.988–1.044
Any Axial subgroup	0.006	1.233	1.062–1.432	0.155	1.023	0.991–1.056
Any Peripheral subgroup	0.038	1.178	1.009–1.375	0.145	1.022	0.992–1.053
Axial-only subgroup	0.071	1.491	0.967–2.299	0.696	1.028	0.895–1.181
Peripheral-only subgroup	0.153	0.298	0.057–1.567	0.949	1.008	0.785–1.295
Axial and Peripheral Overlapped	0.014	1.252	1.047–1.498	0.097	1.030	0.995–1.067

Note: SpA: spondyloarthritis.

**Table 4:** Risk of hepatitis B (HCV) infection associated with longer disease duration in participants with SpA.

Factors & Covariates	Wald	*p-*Value	OR	95% CI for OR
Univariable				
SpA Disease Duration (5-year blocks)	0.021	0.884	0.991	0.880–1.117
Multivariable				
**SpA Disease Duration (5-year blocks)**	**1.430**	**0.232**	**1.108**	**0.937–1.310**
**Delay in SpA Diagnosis**	**0.030**	**0.863**	**0.997**	**0.965–1.031**
Age (year)	1.778	0.182	1.016	0.993–1.039
Gender (reference: Female)	0.446	0.504	1.208	0.694–2.101
Current BMI	0.060	0.807	0.995	0.952–1.039
Smoking (pack-year)	0.156	0.693	0.996	0.976–1.016
Alcohol (reference: Never)	4.956	0.175		
Ex-drinker	0.020	0.887	1.078	0.385–3.021
Current, <3 units/day	0.995	0.319	1.351	0.748–2.443
Current, ≥3 units/day	4.749	0.029	2.793	1.109–7.035
Ever use of NSAIDs	0.452	0.501	1.275	0.628–2.585
Ever use of Steroids	0.727	0.394	0.758	0.401–1.433
Ever use of Synthetic DMARDs	0.069	0.793	1.069	0.648–1.763
Ever use of Biologic DMARDs	1.764	0.184	1.417	0.847–2.368
HLA-B27 positivity	0.473	0.492	0.814	0.453–1.464
Country-based variation	57.906	<0.001		

Note: SpA: spondyloarthritis; BMI: body mass index; NSAID; non-steroidal anti-inflammatory drugs; DMARDs: disease modifying anti-rheumatic drugs; HLA-B27: Human Leucocyte Antigen B27.

**Table 5:** Risk of hepatitis C (HPC) infection associated with longer disease duration in participants with SpA.

Factors & Covariates	Wald	*p-*Value	OR	95% CI for OR
Univariable				
SpA Disease Duration (5-year blocks)	0.076	0.783	0.971	0.790–1.194
Multivariable				
**SpA Disease Duration (5-year blocks)**	**0.330**	**0.565**	**0.926**	**0.713–1.203**
**Delay in SpA Diagnosis**	**0.613**	**0.434**	**0.981**	**0.934–1.030**
Age (year)	17.796	<0.001	1.074	1.039–1.110
Gender (reference: Female)	0.469	0.493	1.338	0.582–3.077
Current BMI	1.699	0.192	0.948	0.875–1.027
Smoking (pack-year)	0.642	0.423	0.985	0.948–1.023
Alcohol (reference: Never)	1.432	0.698		
Ex-drinker	0.014	0.905	1.101	0.224–5.419
Current, <3 units/day	0.915	0.339	0.535	0.148–1.928
Current, ≥3 units/day	0.195	0.658	1.492	0.253–8.792
Ever use of NSAIDs	0.002	0.965	1.031	0.264–4.019
Ever use of Steroids	1.557	0.212	0.543	0.208–1.417
Ever use of Synthetic DMARDs	3.648	0.056	2.699	0.974–7.475
Ever use of Biologic DMARDs	0.869	0.351	1.448	0.665–3.156
HLA-B27 positivity	0.320	0.572	0.777	0.324–1.862
Country-based variation	16.149	0.761		

Note: SpA: spondyloarthritis; BMI: body mass index; NSAID; non-steroidal anti-inflammatory drugs; DMARDs: disease modifying anti-rheumatic drugs.
